# Pathways to honesty: Exploring the ecological desistance of atypical lying features

**DOI:** 10.1111/jora.70130

**Published:** 2025-12-29

**Authors:** Romain Decrop, Emma Rodgers, Scarlet Cho, Alyssa Briones, Jordan Beardslee, Elizabeth Cauffman

**Affiliations:** ^1^ Department of Psychological Science University of California, Irvine Irvine California USA

**Keywords:** adolescence, delinquency, emerging adulthood, lying, parenting, peers

## Abstract

Atypical lying (i.e., dishonesty that is excessive, impulsive, for fun, or lacks clear motive) may signal broader developmental risks. This study examined whether baseline levels and changes in parenting, peer, and individual factors were associated with trajectories of atypical lying from ages 14 to 26. Data from 1354 participants (86.41% male; baseline *M*_age_ = 16.04; 41.5% Black, 33.5% Hispanic, 20.2% White, 4.8% Other) in the Pathways to Desistance study were analyzed. Results indicate that higher risk (parental hostility, antisocial peer influence, and perceived thrill of crime) and lower protective factors (parental warmth, resistance to peer influence, and psychosocial maturity) were associated with more persistently atypical lying scores. Notably, we highlight which factors are most strongly related to the growth of atypical lying and ways to target them through programs aimed to promote honesty. Overall, our findings underscore the role of early contexts and developmental synchrony in atypical lying.

## INTRODUCTION

Lying is a universal behavior that involves the deliberate expression of messages that the communicator does not believe to be true (Hart & Curtis, 2023). Although common throughout development, the emergence and refinement of lying depend on the complex coactions of cognitive processes (e.g., theory of mind, executive functioning; Talwar, 2018). Typically, the behavior increases in both frequency and sophistication from infanthood to early adolescence as youth test their psychosocial abilities and navigate their environments in search of autonomy. Then, as they mature at their own rates, internalize social norms, form moral identities, and adopt more constructive problem‐solving strategies, lying subsides through late adolescence and early adulthood (Decrop & Docherty, 2024). Notably, this developmental pattern is influenced by individual and contextual factors, with those who continue to lie prolifically (i.e., 3–5+ times per day) or in atypical ways (e.g., for fun or no reason) beyond the normative desistance phase being more at risk for a host of negative outcomes in adulthood (e.g., interpersonal difficulties, legal troubles, and substance use; Curtis & Hart, 2022; Decrop & Docherty, 2025). However, there lacks longitudinal research aimed to identify early predictors of problematic lying tendencies across the normative desistance stage of the behavior, particularly among justice‐involved youth. This information is crucial to help inform programs aimed at promoting honesty and reducing deception.

### Atypical lying

Despite broad societal instructions to avoid telling lies, lying is morally ambiguous in nature, as it can serve both prosocial and antisocial utilities (Decrop & Docherty, 2024). For example, while most agree that it is wrong to lie on your taxes for financial gain, many find it acceptable to lie about liking your coworker's new shoes to maintain the relationship. In addition, although people are generally honest and only lie opportunistically when the truth causes a barrier to a desired outcome (Levine, 2020), some who lie excessively often do so impulsively and without a clear motive (Curtis & Hart, 2022). For these reasons, some researchers focus on the problematic dimensions of lying rather than the frequency of all lies. Notably, Decrop and Docherty (2025) modeled change in atypical lying features (i.e., lying excessively, impulsively, for fun or no reason) in youth ages 14–26 and found that most individuals were less likely to endorse atypical features as they aged into adulthood, but that those who maintained elevated levels were at heightened risk for offending and substance use. These results align with existing developmental understandings of lying frequency and the associated negative outcomes (e.g., disruptive behavior; Gervais et al., 2000; Talwar & Crossman, 2011), and they underscore the importance of examining lying beyond just frequency. Nevertheless, it is unlikely that these developmental trajectories are hermetic and not influenced by cooccurring growth from other domains.

Indeed, lying is a social phenomenon, and one that hinges upon interpersonal dynamics. Thus, it is possible that developmental trajectories of lying are influenced by not only individual changes but also interpersonal ones. Albeit a wealth of evidence suggests that adolescents and young adults are impacted by their parent and peer relationships (Irani et al., 2024; Laursen & Veenstra, 2021), it remains understudied how these relationships influence the growth of atypical lying. Similarly, although Decrop and Docherty (2025) found that specific psychopathic traits (e.g., impulsiveness) predicted atypical lying trajectories, prior work has not investigated whether other relevant individual factors (e.g., reward processing) and changes in those factors also influence these trajectories. This is especially consequential for justice‐involved youth because lying can reflect and reinforce broader antisocial behaviors, such as evading accountability, sustaining delinquent peer affiliations, and facilitating offending (Decrop & Docherty, 2024, 2025). In these contexts, such behaviors can complicate supervision, strain relationships with authority figures, weaken court‐ordered programming, hinder rehabilitation and sentencing outcomes, and heighten the risk of continued system involvement (Monahan et al., 2009; Schubert et al., 2004).

Put simply, research assessing broad developmental trends is best supplemented by work that explores how these trends can be modified. Specifically, the transactional model of lying highlights that lying develops through reciprocal interactions between individual and contextual factors, creating feedback loops that reinforce and stabilize deceptive patterns over time (Talwar & Crossman, 2011, 2022). This framework underscores the importance of examining multiple developmental domains to clarify how lying patterns emerge, persist, or change, providing a foundation for targeted efforts to reduce problematic lying and promote honesty. Consequently, this exploratory study aims to extend developmental understandings of lying by examining how a host of social and individual risk and protective factors both predict, and codevelop with, atypical lying features. Using a large sample of justice‐involved youth, we consider how parental hostility and warmth, antisocial peer influence, resistance to peer influence, perceived rewards, and psychosocial maturity are related to the development of atypical lying features during the normative desistance period of the behavior (i.e., adolescence through young adulthood).

### Parenting style

Parenting plays a fundamental role in shaping children's psychosocial development, including their lying tendencies (Bornstein, 2019; Talwar & Crossman, 2022). While parental guidance generally aims to discourage dishonesty, the way parents interact with their children can influence lie telling (Ding et al., 2023; Talwar & Crossman, 2011). Specifically, parental hostility, characterized by harsh discipline, rejection, and emotional or physical aggression, is a known risk factor for externalizing behaviors like lying (Lansford et al., 2010; Trentacosta et al., 2009). This parenting style can erode trust and create an adversarial environment where lying becomes a maladaptive and self‐protective problem‐solving strategy to avoid conflict (e.g., punishment, criticism). Over time, deception could be reinforced by the successful avoidance of hostility, ultimately becoming ingrained as a habitual or reflexive response (Decrop & Docherty, 2024). Conversely, parental warmth, marked by affection, support, and responsiveness, acts as a protective factor against dishonesty by fostering an emotionally secure environment where youth feel comfortable telling the truth, even when it is difficult (Dotterer & Day, 2019; Laible & Thompson, 2000). By promoting open communication and prosocial values, warm parenting can reduce the perceived need for deception (Ding et al., 2023; Talwar & Crossman, 2011).

Nevertheless, parenting research primarily focuses on younger children, and it is unclear whether the risk and protective effects of parental hostility and warmth on lying persist through adolescence and young adulthood as the role of parents change and lying normatively desists. Indeed, as youth gain autonomy, some parents may ease direct monitoring and place more trust in their children while others may respond with stricter control and harsher discipline if conflicts arise around independence and rule breaking. In parallel, warm interactions may either diminish if relationships become more distant or deepen if parents successfully renegotiate their role. Either way, these evolving dynamics theoretically shape how adolescents weigh the costs and benefits of honesty, with greater hostility making lying useful to avoid punishment or conflict and greater warmth reducing the incentive to lie by fostering trust and open communication (Dishion & Patterson, 2006; Talwar & Crossman, 2011). Empirically, studies have found that changes in these parenting styles among justice‐involved adolescents and young adults are linked to changes in antisociality (e.g., offending, psychopathic traits; Backman et al., 2021), but no work has specifically focused on lying. Thus, we should explore both how parental hostility and warmth in adolescence influence the desistance of atypical lying features into adulthood, as well as how change in those parenting styles during that time is associated with the rate at which youth become more honest. However, in doing so, we must consider the influence of individuals' friend groups and their increasing role across adolescence and young adulthood.

### Peer influence

Overall, research on lying and peer influence is scarce, with broader findings suggesting that in group decision‐making contexts, groups tend to be collectively greedier, more dishonest, and more adept at justifying lying as morally defensible than individuals acting alone (Cohen et al., 2009; Kocher et al., 2018). This is partly due to a diffusion of moral responsibility across members that reduces individuals' personal guilt for dishonest behavior (Curtis & Hart, 2022). Worrisomely, dishonesty can be socially contagious, with one study concluding that when a cheater was added to a group, three new cheaters were “created” (Carrell et al., 2008), and another that liars generally socialize with liars and honest people with honest people (Mann et al., 2014). Combined with findings that exposure to lies increases children's lying (Talwar & Crossman, 2022), that youth may be more inclined to start lying in group settings (Decrop & Docherty, 2024), and that peer influence is one of the strongest predictors of antisocial behaviors (Dishion & Patterson, 2006), it is critical to unearth the role of peers in the desistance or maintenance of atypical lying patterns. One way to target this psychosocial process is to examine both an individual's exposure to antisocial peers, as well as their ability to resist that influence.

Indeed, being entrenched in a deviant peer network that regularly invites an individual to commit crimes (i.e., antisocial peer influence) can distort perceptions of norms surrounding transgressions and influence the proper development of moral decision‐making (Dishion & Patterson, 2006; Garner et al., 2008). This is especially pertinent to consider during adolescence, a period marked by peaks in peer orientation (i.e., looking to peers for direction), sensitivity to social reinforcement, and tendencies to conform to peer behaviors (Steinberg, 2008). In addition, peer networks and influence processes also tend to evolve as adolescents form new affiliations, disengage from old ones, and navigate changing group norms. These shifts can alter how deception is modeled, perceived, and rewarded, which in turn can shape whether lying becomes reinforced or discouraged over time (Decrop & Docherty, 2024; Monahan et al., 2009). During this time, susceptibility to peer influence may also amplify tendencies toward deception, particularly in environments where norm‐breaking behaviors are normalized or rewarded. However, as adolescents develop greater autonomy and cognitive maturity, they become more resistant to peer pressure and can avoid making choices or adopting views under the influence of others (Monahan et al., 2009). Although this growth intuitively seems to facilitate the natural desistance of lying (e.g., less likely to lie to fit in), no work has confirmed this or examined how changes in antisocial peer influence and resistance to peer influence codevelop with atypical lying.

### Individual factors

Beyond external influences, individual processes are important to consider given that around 60% of the variance in lying is driven by personality traits and cognitive abilities (e.g., executive skills; Decrop & Docherty, 2024; Serota et al., 2022). Following the rational choice theory, which states that transgressions occur when rewards appear to outweigh the risks (Becker, 1968), individuals who find lying amusing may be doubly rewarded (i.e., enjoyment from the thrill of lying and the practical gains; Decrop & Docherty, 2025). This is particularly pertinent in adolescence, when individuals are prone to sensation‐seeking due to heightened reward sensitivity (Braams et al., 2015). More specifically, while sensation seeking describes a broad and normative developmental tendency, the perceived thrill of crime reflects a more atypical form of reward sensitivity capturing excitement derived from transgressive acts. As such, individuals with elevated perceived thrill of crime may exhibit more persistent or excessive deceptive behaviors, as the intrinsic excitement of breaking social norms compounds the behavior's allure.

Beyond these characteristics, psychosocial maturity may also influence patterns of lying across development. Psychosocial maturity encompasses elements of self‐reliance (i.e., internal control and independent decision‐making), identity (i.e., self‐esteem, clarity of self, and consideration of life goals), and work orientation (i.e., pride in completion of tasks), and grows into adulthood (Cauffman & Steinberg, 2000; Greenberger & Sorensen, 1974). Generally, during this transition, gains in maturity may facilitate greater responsibility (i.e., more consideration for risks), behavioral control, and moral identity formations that limit dishonesty (Decrop & Docherty, 2024; Monahan et al., 2009). However, there is variability in maturation rates among individuals, with some youth reporting more psychosocial maturity than others, and thus, it is plausible that youth who are more psychosocially mature would display different trends in lying across time.

### Current study and hypotheses

This exploratory study investigates how risk (r) and protective (p) factors from parental, peer, and individual domains influence the growth of atypical lying features from ages 14 to 26 in a sample of justice‐involved youth. We first test how parental hostility (r) and warmth (p), antisocial peer influence (r), resistance to peer influence (p), perceived thrill of crime (r), and psychosocial maturity (p) in middle adolescence predict the ensuing latent growth factors of atypical lying. In doing so, we control for exposure to violence, neighborhood conditions, and psychopathic traits, which are disproportionately present in justice‐involved samples and may influence lying by increasing psychosocial risk (Curtis & Hart, 2022; Dierkhising et al., 2013; Sampson & Raudenbush, 1999; Waller et al., 2018). We then examine how developmental changes in our risk and protective factors align with the lying trajectory. Although we would expect each risk and protective factor to be associated with either the maintenance or desistance of atypical lying features, respectively, it is unclear which effects will remain significant when considered simultaneously. The current study contributes to preexisting understandings of how lying develops, and in doing so, advances knowledge to help inform intervention efforts.

## METHODS

### Participants

We examined data from the Pathways to Desistance study, a longitudinal study of 1354 juvenile offenders in Philadelphia, PA (*n* = 700) and Phoenix, AZ (*n* = 654). Consistent with national trends, the sample was predominantly male (*n* = 1170, 86.41%), reflecting the overrepresentation of males in the justice system (Puzzanchera et al., 2022). Nevertheless, given evidence that lying patterns do not systematically differ by sex (Curtis & Hart, 2022), this imbalance is not expected to limit the generalizability of the developmental processes examined. Eligible participants were 14–17 years old and were charged with a felony or serious nonfelony offense. At baseline, the average age was 16 (SD = 1.14) and 41.5% of participants identified as Black, 33.5% as Hispanic, 20.2% as White, and 4.8% as another ethnicity. Since only one participant had a rounded age of 19 at baseline, this participant was excluded from analyses to ensure adequate representation across age groups and to limit model bias, error, and uncertainty.

### Procedures

Researchers approached 2008 youth and 67% agreed to participate. They engaged in two 2‐h interviews at baseline and were followed‐up with every 6 months for a 3‐year period and then annually for 4 years—totaling 11 waves of data across 7 years. Data collection spanned from November 2000 to March 2010 (retention rates across waves: range = 84–94%, M = 90%, range of male participants = 1170–962; range of female participants = 184–172; for full study procedure, see Schubert et al., 2004). Given that participants had rounded ages between 14 and 18 at baseline, age‐based longitudinal analyses allow for investigations that span across 13 years from adolescence to adulthood (ages 14–26). Visit http://pathwaysstudy.pitt.edu/ for study and measure details, with data publicly available at icpsr.umich.edu/web/NAHDAP/studies/29961. This secondary data analysis study was not preregistered and falls under the University of California Irvine's Institutional Review Board (IRB #20141706).

### Measures

#### Trajectory variables

Descriptive information of variables used across waves is in Appendix S1: Table S1.

##### Youth Psychopathy Inventory—Lying Subscale

The Youth Psychopathy Inventory (Andershed et al., 2002) was administered at Waves 2 through 11 and consists of 50, 4‐point Likert items (ranging from 1, “Does not apply well at all” to 4, “Applies very well”). The lying subscale is the sum of five items: “Sometimes I find myself lying without any particular reason,” “Sometimes I lie for no reason, other than because it's fun.” “I've gotten into trouble because I've lied too much.” “I like to spice up and exaggerate when I tell about something,” and “It's fun to make up stories and try to get people to believe them.” As noted in Decrop and Docherty (2025), higher scores indicate more “atypical endorsements of a higher tendency to lie without a clear motive or for one's enjoyment or amusement, as well as an inclination for, or history of, dishonesty (p. 4).” Cronbach's Alpha (*α*) values range from .79 to .82 across waves.

##### Parental Warmth and Hostility

The Quality of Parental Relationships Inventory (Conger et al., 1994) measures perceptions of maternal and paternal warmth and hostility using 42 4‐point Likert scale items (21 per; 1 = “Always” to 4 = “Never”). The warmth subscale uses nine items such as “How often does your mother let you know she cares?” and “How often does your father say he loves you?”, while the hostility subscale uses 12 items in line with “How often does your mother throw things at you?” and “How often does your father get angry at you?” For each caretaker or adult responsible for raising the subject, items were averaged at each wave, with higher scores on the warmth subscale reflecting a more nurturing and supportive parental figure and on the hostility subscale a harsher and more hostile one. However, participants were only administered the questionnaire if they lived with their caretaker, and thus since many no longer did by Wave 8, analyses only included Waves 1–7, using the highest score from either caretaker to assess warmth and hostility (Waves 1–7 *α* range: Maternal Warmth = .92–.93, Paternal Warmth = .95–.96, Maternal Hostility = .79–.85, and Paternal Hostility = .78–.88).

##### Antisocial Peer Influence

The Antisocial Influence subscale of the Peer Delinquent Behavior scale (Thornberry et al., 1994) includes seven 5‐point Likert scale items (1 “None of them” to 5 “All of them”) that assess the degree to which one's peers influence or try to influence them to act antisocially (e.g., “During the last six months how many of your friends have suggested that you should sell drugs?”). A higher mean score at each wave indicates more exposure to antisocial influence (Waves 1–11 *α* range = .89–.94).

##### Resistance to Peer Influence

The 10‐item Resistance to Peer Influence scale (Steinberg, 2000) measures how much adolescents value peer opinions. Each item presents two opposing scenarios (e.g., “Some people go along with their friends to keep them happy” vs. “Others refuse, even if it upsets their friends”), followed by a truth rating (“sort of true” or “really true”). Participants first choose the statement that best describes them, then indicate how true it is for them. This two‐step response format captures both the direction of participants' tendencies (i.e., resist or yield to peer influence) and the strength of those tendencies (i.e., how accurate that description feels to them). Items are scored from 1 to 4, with higher mean scale scores at each wave indicating greater autonomy and resistance (Waves 1–11 *α* range: .73–.78).

##### Perceived Thrill of Crime

The Personal Rewards of Crime scale (Nagin & Paternoster, 1994) uses seven items to assess youths' perceived thrill for criminal activities (e.g., “How much thrill or rush is it to break into a store or home?”; 0 = “no fun at all” to 10 = “a great deal of fun”). A total mean score was computed at each wave, with higher values indicating greater perceived excitement or fun for transgressing (Waves 1–11 *α* range = .88–.91).

##### Psychosocial Maturity

The Psychosocial Maturity Inventory (PSMI; Greenberger & Sorensen, 1974) assesses self‐reliance, identity, and work orientation using 30 items (10 per subscale) on a 4‐point Likert scale (1 = “Strongly Agree” to 4 = “Strongly Disagree”). Self‐reliance measures internal control and decision‐making (e.g., “Luck decides things for me”), identity assesses self‐esteem, clarity, and life goals (e.g., “I change how I feel and act so often that I sometimes wonder who the ‘real’ me is”), and work orientation captures pride in task completion (e.g., “I hate to admit it, but I give up on my work when things go wrong”). A mean score was computed with higher scores indicating more responsible behaviors (Waves 1–11 *α* range = .89–.93).

#### Baseline controls

##### Psychopathic traits

This study adapted the Psychopathy Checklist: Youth‐Version (PCL‐YV; Forth et al., 2003) to assess psychopathic traits. In addition to examining the participants' responses from items in the original PCL‐YV open‐ended interview, trained researchers included court records and parent collateral interviews to complete a 20‐item PCL‐YV rating form that uses a 3‐point Likert scale (0 = “does not apply,” 1 = “applies somewhat,” and 2 = “fully applies”). A total sum score was computed with higher values indicating more psychopathic traits (Wave 1: M = 15.91, SD = 7.74, range: 1–39, *N* = 1299).

##### Exposure to violence

The Exposure to Violence Inventory (Selner‐O'Hagan et al. 1998) was slightly modified in this study and includes six self‐report items assessing violence directly experienced (e.g., “Have you ever been shot at?”) and seven items assessing violence that was witnessed (e.g., “Have you ever seen someone else get attacked with a weapon?”). Since the scales moderately overlap (*r* = .54, *p* < .001), like Waller et al. (2018), a sum of the 13 dichotomous items (0 = *No*, 1 = *Yes*) was used. Higher scores indicate more witnessed or experienced violence (Wave 1: M = 5.34, SD = 2.99, *N* = 1350).

##### Neighborhood disorder

The Neighborhood Condition Measure is adapted from Sampson and Raudenbush (1999) and assesses physical and social disorder surrounding adolescents' homes. The two disorder domains are highly correlated (*r* = .83, *p* < .001), and thus the total mean of all 21 self‐reported 4‐point Likert scale items (1 = “Never,” 4 = “Often”) is used (e.g., “How often are adults fighting or arguing loudly within your neighborhood?” “How often are there needles or syringes within your neighborhood?”). Higher scores indicate a risk for worse neighborhood conditions and more disorder (Wave 1: M = 2.35, SD = 0.75, *N* = 1353).

##### Demographic information

Three dichotomous race/ethnicity variables (0 = *No*, 1 = *Yes*) were created for White, Hispanic, and other participants, making Black individuals the reference group. Similarly, sex was measured as a binary variable (0 = Female, 1 = Male). See Participants section for demographic breakdown at baseline.

### Analytical plan

Analyses were conducted in *Mplus* (Muthén & Muthén, 1998–2017). We first examined bivariate Pearson correlations to determine whether atypical lying was associated with the risk and protective factors at each wave. Then, we evaluated the longitudinal measurement invariance of the risk and protective factors, as well as atypical lying, to verify that changes in scores across time reflect developmental processes rather than shifts in item functioning. We compared configural, metric, and scalar models and considered invariance supported when the change in fit indices was within thresholds (ΔCFI, ΔTLI, ΔSRMR ≤ .010; ΔRMSEA ≤ .015; Chen, 2007).

Next, we identified the univariate latent growth curve trajectories of all analysis variables with unconditional latent growth curve models (Curran & Hussong, 2003) and while using all available data: atypical lying (Waves 2–11), parental hostility and warmth (Waves 1–7), antisocial peer influence, resistance to peer influence, perceived thrill of crime, and psychosocial maturity (all Waves 1–11). We determined the best fitting growth trajectories for each construct by comparing sequential models that differed in how they characterized change over time (i.e., intercept‐only, linear, and quadratic fixed and random effects). The best‐fit models were chosen based on lower relative AIC, BIC, and SABIC indices, significant chi‐square difference tests of nested models, and considerations of parsimony and interpretability. Indeed, beyond improved fit, more complex models were only retained when those growth factors offered meaningful information about change over time (i.e., were significant; Curran & Hussong, 2003).

Notably, we used a robust maximum likelihood estimator (MLR) and the “tscore” option to create definition variables with age as the individually varying time of observation by wave (i.e., aligning data by age rather than wave; Mehta & Neale, 2005). This allows us to overcome the limited overlaps in some measures across waves (e.g., parenting and lying variables only overlap for Waves 2–7) by leveraging the study design where participants ranged in age at each wave. As such, we were able to model the growth of atypical lying from ages 14 to 26, the growth of the parenting variables from ages 14 to 21, and the remaining risk and protective factor growths from ages 14 to 26. The age variables were centered at 20, the most populated age for the core lying variables of interest, to aid in model convergence by reducing multicollinearity between intercept and slope factors and bringing parameter estimates closer to their means. Centering the age variables at age 20 for all models also allowed the unconditional growth curve and parallel process models to remain consistent with each other (i.e., intercept at 20 for each).

In the following step, we conducted our baseline predictor model to examine the extent to which the risk and protective factors at baseline were related to mean levels (intercept) and change over time (slope) of atypical lying. In this model, we used the best fitting unconditional growth model for atypical lying and regressed the latent growth factors (e.g., intercept; slope) on baseline levels of all the risk and protective factors (parental hostility and warmth, antisocial peer influence, resistance to peer influence, perceived thrill of crime, and psychosocial maturity) and control variables (psychopathic traits, exposure to violence, neighborhood condition, sex, and race/ethnicity). All predictors and controls were set to correlate with each other.

In the final step, we ran a parallel process model to examine how atypical lying codevelops with the risk and protective factors across adolescence and early adulthood. This model simultaneously included the best fitting unconditional growth curves for each risk and protective factor, as well as those of atypical lying, using all available data. In doing so, we can examine the interfactor correlations between the risk and protective factors and atypical lying while statistically accounting for the effects of the other factors. Specifically, this allows us to examine the associations of the: (1) intercept–intercept growth factors that reflect how constructs relate at the centered age of 20; (2) intercept–slope growth factors that indicate whether levels of one construct at the centered age of 20 are linked to the rate or direction of change in another; (3) slope–slope growth factors that capture the extent to which trajectories covary over time (e.g., whether increases in one domain correspond to similar changes in another); (4) slope–quadratic growth factors, which reflect whether linear change in one construct is associated with acceleration or deceleration in another; and (5) quadratic–quadratic growth factors, which capture how the degree and shape of acceleration or deceleration covary in constructs across time.

Given the computational intensity, we did not include the baseline controls in the parallel process model (e.g., psychopathic traits). Interested readers are encouraged to examine the baseline predictor model results that include them. Additionally, while this model ecologically maps the cooccurring development of atypical lying with parent, peer, and individual domains, the shorter window for parenting variables due to limited data availability (ages 14–21) may constrain the comparability of growth factors by underestimating associations into adulthood and obscuring later developmental shifts. Nevertheless, since our first model more robustly predicts the intercept factor of lying due to its temporal ordering (i.e., using baseline predictors with controls rather than examining intercept factor correlations that were centered at age 20), the substantive focus of the parallel process model is on the associations between growth factors.

## RESULTS

### Pearson correlations and invariance across waves

Descriptive information of variables used across waves is in Appendix S1: Table S1, with Appendix S2: Table S4 depicting the Pearson correlation coefficients of the lying variables with each risk and protective factor at each interview wave (correlations between risk and protective factors are available upon request). In general, atypical lying is significantly correlated with each variable across waves, with particularly strong relationships at the same waves indicating positive relationships with risk and negative relationships with protective variables. Next, atypical lying, parental warmth and hostility, resistance to peer influence, and psychosocial maturity exhibited full invariance, indicating that observed changes can be interpreted as true developmental shifts rather than measurement artifacts (see Appendix S2: Table S5). However, antisocial peer influence and perceived thrill of crime showed mixed evidence of invariance, with some fit indices (e.g., TLI, SRMR) remaining within acceptable ranges but others exceeding recommended thresholds (e.g., ΔRMSEA = .028 for peer influence; ΔCFI = .028 for thrill of crime). This suggests that changes for these constructs should be interpreted with some caution as some youth may differently interpret the constructs over time.

### Unconditional latent growth trajectories

The fit statistics of the intercept‐only, linear, and quadratic unconditional latent growth trajectories, as well as the best‐fit models' growth factors' covariances, for each variable are in Appendix S2: Tables S6 and S7, with visual depictions in Appendix S2: Figures S1–S7.

#### Atypical lying features

Quadratic growth best fit the data and, on average, individuals at age 20 had an atypical lying feature score of 7.73 (SE = 0.06, *p* < .001). This score decreased from ages 14 to 26 (M = −0.22, SE = 0.01, *p* < .001), though the decrease had deceleration (i.e., less decrease) over time (M = 0.02, SE = 0.01, *p* < .001). Additionally, there was significant variance in the starting values, growth, and deceleration of individuals across time (all *p* < .001), suggesting significant sample heterogeneity in the lying trajectory and that some participants had lower or higher than average starting values and change in scores (e.g., some maintained more elevated levels).

#### Parenting styles

Despite the quadratic models having marginally better fit indices than the linear ones, the linear hostility growth factor and the linear and quadratic warmth growth factors of the quadratic models were not significant. However, all growth factors of the linear models were significant and thus we prioritized model parsimony and retained the linear models. For both styles, on average, participants at age 20 had parental hostility and warmth scores of 1.34 (SE = 0.01, *p* < .001) and 3.07 (SE = 0.02, *p* < .001), respectively, and those scores decreased (Hostility: M = −0.06, SE = 0.01, *p* < .001; Warmth: M = −0.04, SE = 0.01, *p* < .001). There was also significant variability in starting values and growth of both trajectories (all *p* < .001). Of note, given that these variables were not missing at random (i.e., participants were asked items if they lived with a caretaker), we also ran these models without centering the age variable to ensure similar growth. Despite coverage concerns at age 20, post hoc analyses revealed similar growth curves for both centered and noncentered age variables (i.e., intercept at age 20 vs. 14).

#### Peer influence

Quadratic growth best fit the data for both peer variables. Individuals had an average antisocial peer influence score of 1.43 at age 20 (SE = 0.01, *p* < .001) that was decreasing (M = −0.02, SE = 0.00, *p* < .001), though with deceleration (M = 0.01, SE = 0.00, *p* < .001), from ages 14 to 26. Conversely, participants' average resistance to peer influence score at age 20 was 3.35 (SE = 0.01, *p* < .001) and it was increasing during that time (M = 0.06, SE = 0.00, *p* < .001). However, there was also significant deceleration in that increase (M = −0.01, SE = 0.00, *p* < .001). All growth factors, except the quadratic component of the antisocial peer influence trajectory (*p* = .063), had significant variance (all *p* < .001).

#### Individual factors

Quadratic growth best fit the data for both individual factors. On average, individuals' perceived thrill of crime scores at age 20 was 1.60 (SE = 0.05, *p* < .001) and decreasing across ages (M = −0.11, SE = 0.01, *p* < .001), though with deceleration (M = 0.02, SE = 0.00, *p* < .001). Alternatively, the average psychosocial maturity score at age 20 was 3.24 (SE = 0.01, *p* < .001), and was increasing (M = 0.03, SE = 0.00, *p* < .001) with deceleration (M = −0.01, SE = 0.01, *p* < .001) into adulthood. All growth factors had significant variance (all *p* < .001).

### Baseline predictors of the atypical lying trajectory

The regression results of Wave 1 predictors' impact on the latent growth factors of lying features across ages 14–26 are in Appendix S1: Table S2 and depict which factors may impede the desistance of atypical lying. Overall, at baseline (~16 years old), all nonparenting variables were significantly predictive of the atypical lying intercept. Specifically, higher antisocial peer influence (*std est* = 0.18, SE = 0.09, *p* = .048) and perceived thrill of crime (*std est* = 0.10, SE = 0.03, *p* = .001), as well as lower resistance to peer influence (*std est* = −0.28, SE = 0.11, *p* = .012) and psychosocial maturity (*std est* = −0.83, SE = 0.14, *p* < .001), predicted higher atypical lying scores at age 20. Notably, parameter comparisons found psychosocial maturity to be the strongest predictor (*p* < .001). Next, those with higher parental hostility scores were more likely to experience less of a decrease in their lying scores across ages 14 to 26 (slope factor *std est* = −0.09, SE = 0.03, *p* = .002). Similarly, those with lower resistance to peer influence were more at risk to maintain elevated lying scores through flatter decreases (slope factor *std est* = 0.06, SE = 0.02, *p* = .011) and more deceleration in those decreases (i.e., steeper plateaus; quadratic factor *std est* = −0.02, SE = 0.01, *p* = .011). Lastly, white and female participants were more likely to have higher intercept scores than black and male participants, though the sex results should be interpreted cautiously given the majorly male sample.

### Parallel process model of the atypical lying trajectory

We then examined how changes in our risk and protective factors are related to changes in lying trajectories. Broadly, we found that the lying growth factor associations follow a similar pattern as the baseline predictor model (see Appendix S1: Table S3), with remaining nonlying associations (e.g., parent–peer associations) available upon request. Indeed, at age 20, all risk and protective factor intercepts were significantly associated with the atypical lying intercept, such that higher lying scores were associated with higher risk factor scores and lower protective factor scores. Notably, the perceived thrill of crime scores had the strongest association with atypical lying (Intercept *std est* = 1.51, SE = 0.13, *p* < .001), likely due to the YPI subscale capturing the amusement of engaging in lying which can be viewed as a minor transgression. Additionally, participants with higher atypical lying intercepts at age 20 were more likely to experience greater decreases in parental warmth across time (slope factor *std est* = −0.05, SE = 0.02, *p* < .001). This finding could represent how lying disrupts trust, with caretakers moving away from parental warmth due to being persistently lied to or warm parenting not leading to desired changes.

The slope–slope growth factor associations revealed that participants who reported smaller decreases in lying scores from ages 14 to 26 also showed less positive change in other domains, maintaining higher risk and lower protective factors across time, except for parental warmth which was not significant. Specifically, youth exposed to more consistently high parental hostility into young adulthood were also more likely to maintain elevated atypical lying scores, implying that continued exposure to harsh parenting may hinder normative desistance. Similarly, more stunted growth in psychosocial maturity and resistance to peer influence were associated with more persistent atypical lying, suggesting that underdeveloped self‐regulation and autonomy, as well as a continued difficulty asserting independence from peer pressure, contribute to sustained dishonesty. Finally, smaller declines in the thrill of crime and antisocial peer influence were also linked to higher lying scores over time, indicating that ongoing attraction to deviance and deviant peer contexts may reinforce deceptive behavior. These findings suggest that when protective development is limited and risk exposure remains high, youth are less likely to exhibit typical declines in atypical lying across the transition from adolescence to adulthood.

Furthermore, the only linear growth factors significantly associated with the deceleration in lying trajectories (i.e., slope‐quadratic growth factors) were those depicting changes in parenting styles. Specifically, although both hostile and warm parenting decreased over time, steeper declines in parental hostility were associated with less deceleration in lying trajectories (i.e., more steady decreases), while steeper declines in parental warmth were associated with greater deceleration in lying trajectories (i.e., more plateauing). Beyond parenting, the quadratic growth factors of peer and individual domains were also significantly related to quadratic lying factors. Youth who exhibited less decrease in lying through earlier or steeper plateauing also tended to have higher risk and lower protective scores during this period due to simultaneous decelerations in these domains. In particular, earlier plateauing in thrill‐seeking and antisocial peer exposure was positively associated with earlier plateauing or slower declines in lying, whereas earlier plateauing in psychosocial maturity and resistance to peer influence was negatively associated with lying deceleration, reflecting less pronounced declines over time. Together, this pattern suggests that nonlinear changes in lying were closely synchronized with nonlinear shifts in developmental risk and protective factors, indicating that when atypical lying stopped changing, so did the surrounding processes that sustain or mitigate it.

## DISCUSSION

This study advances developmental understandings of lying by exploring how atypical lying features from adolescence to young adulthood are shaped by, and cooccur with, a host of psychosocial factors from peer, parent, and individual domains. First, we found that atypical lying declined from ages 14 to 26 with deceleration that levels off by participants' late twenties, but that there was significant variability in individuals' starting scores and rates of change. This is consistent with Decrop and Docherty (2025), who identified three distinct developmental trajectories with the males of this sample: a large low, stable group (55.3%), a moderate group with gradual desistance (39.3%), and a small group that maintained elevated lying scores across time (5.4%). Notably, the authors found that the moderate and elevated groups were at increased risk for later externalizing behaviors and thus this heterogeneity underscores the need to understand what contributes to the persistence versus desistance of lying over time. Accordingly, we attempted to identify risk and protective factors in middle adolescence that impede the normative desistance of atypical lying into adulthood and examined how changes in those factors correlated with the lying trajectory. This information helps guide prevention and intervention efforts aimed at promoting honesty by both highlighting characteristics that may place youth at risk of becoming prolific liars and providing recommendations for specific target areas across individuals' lives.

### Baseline predictors of the atypical lying trajectory

We found that 14–18‐year‐old justice‐involved youth who experienced harsher or more violent parenting spent more time with friends who encourage wrongdoing, struggled to stand up to peer pressure, saw transgressions as more exciting, and lacked maturity skills, were more likely to exhibit atypical lying features through adolescence and young adulthood. In addition to broadly targeting these issues prior to middle adolescence, our findings suggest that psychosocial maturity may be an important target of honesty programs as it was the strongest predictor of atypical lying at age 20 in our model. Indeed, youth at baseline who took less personal responsibility and were less orientated toward the future were more likely to engage in persistently elevated patterns of atypical lying across the entirety of the study period. However, when youth are more able to take personal responsibility for their actions and can effectively weigh the long‐term consequences of their actions, they are less likely to lie in a way that is impulsive and persistent. Along this line, future works will need to investigate whether specific dimensions of psychosocial maturity place individuals more at risk for atypical lying than others.

Additionally, both parental hostility and resistance to peer influence were significant predictors of changes in atypical lying. Thus, while lower psychosocial maturity indicates a more elevated trajectory intercept and thus trajectory (i.e., overall higher lying scores), the latter two more directly impact the rate at which atypical lying scores decrease across time. In other words, while psychosocial maturity may simply lead to a delayed desistance in atypical lying (i.e., low psychosocial maturity predicts a greater starting point and, therefore, a longer time until a nonatypical threshold is passed), greater parental hostility and lower resistance to peer influence may impede the desistance altogether and maintain elevated atypical features of lying beyond normative periods. Specifically, youth with parents who used harsher communication styles reported less of a decrease in atypical lying behavior across the study period, and youth who were less resistant to peer influence displayed a similar pattern. These results underscore the long‐term importance of interpersonal relationship dynamics in the lives of justice‐involved youth. Additionally, our findings suggest that these effects span across relationship types, such that both parents and peers are relevant to the trajectories of lying.

### Parallel process model

We then found that lying develops in tandem with changes in parenting style, peer context, and personal functioning. Specifically, youth with more atypical lying features at age 20 concurrently reported higher parental hostility, antisocial peer influence, and thrill of crime, along with lower parental warmth, resistance to peer influence, and psychosocial maturity. Overall, these patterns align with prior research. For example, findings on parental hostility and warmth align with family process research suggesting that even in late adolescence, shifting parental dynamics (e.g., becoming less punitive and more supportive) can spur positive changes in youth behavior (Laible & Thompson, 2000). In our study, steeper decreases in hostility predicted more sustained lying declines due to less deceleration in growth, which may reflect improvements in family climate that help maintain desistance. In contrast, steeper decreases in warmth were associated with earlier plateauing in growth, likely reflecting reductions in parental support as adolescents transition toward greater autonomy. This pattern highlights that hostility and warmth play distinct roles in shaping the developmental timing of change in lying.

Next, perceived thrill of crime emerged as the strongest concurrent correlate of lying at age 20, underscoring a reward‐based motivation for dishonesty in some youth (Decrop & Docherty, 2025). Whereas lying is often conceptualized as a strategic tactic, for certain individuals, it can also be intrinsically gratifying, heightening the challenge for interventions that rely solely on deterrence. This finding aligns with theories emphasizing risk–reward appraisals in deception, as the same mechanisms that make transgression feel thrilling may also heighten the perceived rewards of lying (Becker, 1968; Decrop & Docherty, 2025). However, the consequences of getting caught lying are generally less severe than those of criminal activities, and thus, this thrill may more easily override any perceived punishments for lying in leading to problematic tendencies. This is especially informative for prevention and intervention efforts, which should focus on reducing the perceived thrill of lying by raising awareness regarding the seriousness of lying's consequences. As such, future studies will want to explore the similarities and differences between atypical lying and the thrill of antisocial behavior, and their differing relations with perceived punishments, to identify underlying mechanisms leading to broader dysfunction.

### Implications

This study highlights the value of utilizing an ecological and developmental framework to understand atypical lying from adolescence to adulthood. While we did not focus on direct lie telling, our measure captures the cognitive, emotional, and motivational features that underlie prolific deception and extend beyond opportunistic acts (Decrop & Docherty, 2025). As such, we likely captured more serious and stable lying traits than would frequency alone. This means that, although self‐reported dishonesty is associated with behavioral measures of lying (Curtis & Hart, 2022), our effects may have been smaller and less malleable than if we had directly analyzed lie telling. Nevertheless, all the factors we examined demonstrated some relation to atypical lying, with some predictors being particularly impactful. Thus, in addition to broadly targeting problematic lying across parent, peer, and individual domains, programs aimed to reduce lying will also need to appropriate extra resources toward targeting psychosocial maturity, parental hostility, resistance to peer influence, and perceived thrill of crime. Given atypical lying's malleability and its association with these factors, taking a holistic approach that focuses these components concurrently may be a promising avenue toward efficiently promoting honesty.

This aligns with the transactional model of lying, which emphasizes interactions between individual and contextual factors and supports interventions that target multiple domains to disrupt the feedback loops sustaining deceptive behavior (Talwar & Crossman, 2011, 2022). Fortunately, several existing intervention frameworks can provide strategies for targeting these factors. For example, Multisystemic Therapy and Functional Family Therapy address parental hostility and peer influence through family counseling and psychosocial skill‐building. These approaches can be integrated with cognitive‐behavioral components of Aggression Replacement Training, which teaches impulse control, moral reasoning, and prosocial decision‐making, and could further strengthen psychosocial maturity and decrease the perceived thrill of antisocial behaviors like lying. Additionally, adapting motivational interviewing techniques to challenge reward perceptions and reinforce intrinsic motivations for honesty might enhance youths' personal accountability and their openness to behavioral change. Collectively, integrating these evidence‐based strengths would offer a cohesive and comprehensive strategy for effectively mitigating atypical lying and fostering honesty among adolescents.

### Justice‐system involvement

Following normative developmental trends, our participants, on average, reported decreases in lying tendencies from adolescence to adulthood. Although more direct trajectory comparisons with community samples are needed, a justice‐involved sample is well suited to explore our topic given their generally higher risk for adverse experiences and outcomes (Bergquist et al., 2022; Dierkhising et al., 2013; Folk et al., 2021). This not only allows for more variability in minor transgressions (e.g., lying) and predictors of risk than would be found in the community (i.e., higher base rates), but it also directly focuses on a population that is already in need of intervention services. Indeed, this is especially relevant given that justice‐system involvement can uniquely shape our predictors across the parenting, peer, and individual domains. In other words, justice‐involved youth may experience compounded developmental disruptions, influencing how and why lying emerges and persists.

In terms of parenting, justice‐involved youth who experience greater and/or longer exposure to punitive environments (e.g., jail) may have less positive parent–child interactions. For example, a mother may struggle to build a warm and secure relationship while her child is incarcerated, or perhaps a father may become more hostile due to the stigmas and strain of justice system involvement (e.g., viewing their child as less trustworthy and more of a financial stressor). Similarly, justice‐involved youth are at heightened risk to be embedded within antisocial peer networks, whether voluntarily through shared deviant interests (e.g., substances) or systematically through institutional placement (e.g., secure confinement, disruptive behavior programs). On an individual level, youth involved in the justice system may have more internal motivation to use lying to avoid consequences than youth who are not since they may face steep consequences for behaviors that are considered normative in other populations of youth (i.e., substance use experimentation is not the “first strike”).

Interestingly, parental warmth emerged as one of the least influential variables for our models (e.g., only nonsignificant predictor, only nonsignificant slope factor associations) despite being correlated with atypical lying across waves (*p* < .05 at and across waves). This suggests that when considered alongside other risk and protective factors, warmth alone may not create a sufficiently secure and nurturing environment for adolescents to either learn appropriate uses of dishonesty or discourage atypical lying through open communication. Notably, warmth was also the only protective factor that declined over time, which may reflect cultural norms that offer less warmth to adolescent and young adult males, or the broader disruptions tied to justice‐system involvement. Since justice‐involved youth often face cumulative risks, these adolescents may require an excess of warmth to counteract risk influences and foster honest communication and problem‐solving; however, the downward trend in warmth suggests they may never reach levels sufficient to offset those risks. Alternatively, parental warmth might be more pivotal earlier in childhood, before the typical desistance in lying begins. Either way, future research needs to pinpoint how and when warmth best promotes healthy lying development.

### Strengths, limitations, and future studies

We examined 11 waves of data from a large longitudinal study of justice‐involved youth to explore the etiology of atypical lying features across the normative desistance phase of the behavior (i.e., ages 14–26). By incorporating a host of risk and protective factors across varied domains, our ecological study serves as a steppingstone to better understanding lying desistance or persistence into adulthood. Specifically, we highlighted the need for intervention and prevention programs to target multiple levels of individuals' lives and pointed to specific factors that may require extra focus to induce the normative desistance of atypical lying features. This allowed us to make specific recommendations aimed at reducing problematic lying tendencies found to have detrimental outcomes. Nevertheless, our study is not without limitations.

First, our findings will need to be generalized beyond justice‐involved youth from two US cities, as they may not be reflective of other samples (e.g., non‐US, community). Indeed, while it is useful to study atypical lying in justice‐involved youth given their higher likelihood to commit minor transgressions like lying and to have a history of risk factors (i.e., higher base rates and variabilities for both), those with more protective factors may have differing lying trajectories. Additionally, our sample is disproportionately male, which reflects long‐standing structural disparities in justice system contact, with males being more likely to be arrested (Puzzanchera et al., 2022). Given the small female sample size, we were unable to converge a female‐only parallel process model to compare the sexes. Consequently, our results likely capture patterns of lying and related risk most characteristic of male youth, who tend to show earlier onset and higher rates of antisocial behaviors, as well as stronger peer deviance effects than their female counterparts (Dishion & Patterson, 2006; Moffitt, 1993). As such, these findings may not generalize to nonmale youth, whose developmental pathways, social contexts, and mechanisms underlying lying may differ in timing and expression. Furthermore, our study was limited to ages 14–26, and thus we could not account for the effects of our predictors earlier in childhood nor how lying continues to grow through adulthood. Future research will want to examine lying's growth across more diverse samples from different backgrounds and across other developmental stages to best understand how lying can grow differently across individuals.

Second, the focus on atypical lying (e.g., lying for fun or no reason) fails to capture all forms of dishonesty, thus restricting generalization to more strategic, prosocial, or situationally driven lies. For example, it is likely that our predictors may be differently related to prosocial lies meant to maintain relationships, self‐protective lying learned as a maladaptive trauma response, lying to evade law enforcement, or lying due to a broader cultural distrust of authority. However, examining atypical lying is important to consider given that lying is opportunistically based and that justice‐involved youth may have limited social experiences if incarcerated (e.g., solitary confinement). Therefore, future studies may want to differentiate the etiologies of different lying types across samples, as their underlying causes and etiologies may impact the treatment approaches (e.g., focus on trauma, impulse control, perspective taking, and/or interpersonal schemas). Understanding these distinctions will be essential for developing precise interventions tailored to the unique developmental contexts and needs of diverse youth populations.

Furthermore, although longitudinal models improve developmental inferences, the parallel process model provides correlational results and prevents claims about causality or directionality. Indeed, it is possible that lying and the risk and protective factors exhibit bidirectional growth effects (i.e., predict each other), which would synergistically contribute to harmful developmental patterns. Future studies should test more causal models to assess various relationships preceding or resulting from atypical lying features. Moreover, our parenting variables were only examined at seven waves, which reduces the ages simultaneously covered by both the parenting (ages 14–21) and lying trajectories (ages 14–26), thus limiting interpretability across the full developmental spectrum and into later adulthood.

Additionally, we did not analyze any moderation or mediations, and thus we do not know whether these variables are intertwined with each other and leading to mitigating or amplifying effects. Next, although controlling for multiple covariates (e.g., exposure to violence) improved our model specificity, other factors, such as biological markers, school environment, or the extent of justice system contact, may also shape lying trajectories. Along these lines, parent and peer lying behaviors, as well as the quality of these interpersonal relationships, may add nuance to our findings. Finally, we did not examine trends in other cooccurring antisocial behaviors (e.g., offending, substance use) and thus could not determine whether trends in atypical lying were a result of covering up other illicit behaviors. This is particularly relevant for the thrill of crime, which may drive both greater engagement in antisocial acts and the need to conceal those acts through lying, making it difficult to disentangle whether lying reflects intrinsic enjoyment or a functional response to increased offending. As such, future research will want to further explore these factors, as well as others, to help complete our understanding of lying's growth.

## CONCLUSION

This exploratory study offers a nuanced view of atypical lying during a critical developmental window (i.e., desistance stage of lying), revealing how specific risk and protective factors from parent, peer, and individual domains predict and align with the growth of atypical lying features. By integrating both prediction and codevelopment, our findings underscore that lying is not solely the product of early risk, nor is it purely a transient adolescent phenomenon. Rather, it appears to reflect ongoing changes in relationships and psychosocial growth. Based on these results, in addition to broadly targeting problematic lying across parent, peer, and individual domains, programs aimed to reduce lying will also need to specifically target individuals' psychosocial maturity, parental hostility, resistance to peer influence, and perceived thrill of crime. Taken together, results from this exploratory analysis may serve as a crucial first step in identifying the complex ways in which lying behavior is shaped from adolescence to adulthood. As contextual, interpersonal, and individual‐level factors were identified as developing in tandem with lying desistance, future studies may further specify the nature of these relationships and/or critical periods for intervention opportunity.

## AUTHOR CONTRIBUTIONS

All listed authors should have contributed to the manuscript substantially and have agreed to the final submitted version. Romain Decrop worked on the conceptualization, methodology, investigation, formal analysis, and writing (original and reviewing) of this paper. Emma Rodgers, Scarlet Cho, and Alyssa Briones helped write the original draft and review/edit later drafts. Dr. Jordan Beardslee and Dr. Elizabeth Cauffman supervised the process and reviewed/edited later drafts.

## FUNDING INFORMATION

No funding was received for this secondary data analysis.

## CONFLICT OF INTEREST STATEMENT

The authors have no conflicts of interest to disclose.

## ETHICS STATEMENT

The University of California, Irvine's Institutional Review Board approved the study on August 15, 2023 (IRB #20141706).

## PATIENT CONSENT STATEMENT

Parental or participant consent was obtained when data was originally collected. This consent included permission for researchers to later conduct secondary data analyses like the current study. Information on the study procedures and measures can be found in Schubert et al. (2004) or at http://pathwaysstudy.pitt.edu/.

## Supporting information


Appendix S1.

## Data Availability

The data that support the findings of this study are openly available in ICPSR at https://www.icpsr.umich.edu/web/NAHDAP/studies/29961.
