# Supplementary material for: Pathways to honesty: Exploring the ecological desistance of atypical lying features
Source: J Res Adolesc. 2025 Dec 29;36(1):e70130. doi: 10.1111/jora.70130 (PMC12746536; doi:10.1111/jora.70130)
Supplement: Supplementary file 1 — Appendix S1. [file JORA-36-0-s001.docx]

**APPENDIX S1: TABLES**

| Table S1 | | | | | | | | | | | | | | |
| --- | --- | --- | --- | --- | --- | --- | --- | --- | --- | --- | --- | --- | --- | --- |
| *Descriptive Information of Variables Across Waves* | | | | | | | | | | | | | | |
|  | Lying Features | | Parental Hostility | | Parental Warmth | | Peer Influence | | RPI | | Perceived Thrill | | PSMI | |
|  | (Range 5-20) | | (Range 1-4) | | (Range 1-4) | | (Range 1-5) | | (Range 1-4) | | (Range 0-10) | | (Range 1-4) | |
|  | M (SD) | N | M (SD) | N | M (SD) | N | M (SD) | N | M (SD) | N | M (SD) | N | M (SD) | N |
| W1 |  |  | 1.70 (0.49) | 1,332 | 3.27 (0.66) | 1,332 | 1.77 (0.85) | 1,338 | 2.99 (0.58) | 1,345 | 2.36 (2.42) | 1,352 | 3.02 (0.46) | 1,345 |
| W2 | 8.97 (3.36) | 1,077 | 1.45 (0.37) | 1,165 | 3.23 (0.70) | 1,166 | 1.51 (0.73) | 1,242 | 3.07 (0.57) | 1,257 | 2.28 (2.49) | 1,260 | 3.05 (0.46) | 1,257 |
| W3 | 8.48 (3.27) | 1,259 | 1.46 (0.39) | 1,100 | 3.19 (0.68) | 1,100 | 1.47 (0.70) | 1,253 | 3.13 (0.58) | 1,257 | 2.15 (2.40) | 1,259 | 3.12 (0.47) | 1,259 |
| W4 | 8.32 (3.23) | 1,225 | 1.42 (0.35) | 1,045 | 3.16 (0.71) | 1,045 | 1.48 (0.72) | 1,213 | 3.16 (0.56) | 1,223 | 1.99 (2.36) | 1,226 | 3.15 (0.48) | 1,223 |
| W5 | 8.59 (3.24) | 1,224 | 1.43 (0.37) | 1,017 | 3.16 (0.71) | 1,017 | 1.48 (0.74) | 1,222 | 3.19 (0.56) | 1,225 | 1.98 (2.36) | 1,228 | 3.13 (0.49) | 1,225 |
| W6 | 8.08 (3.00) | 1,228 | 1.41 (0.36) | 1,005 | 3.13 (0.74) | 1,005 | 1.41 (0.67) | 1,214 | 3.25 (0.56) | 1,229 | 1.74 (2.30) | 1,231 | 3.18 (0.49) | 1,229 |
| W7 | 8.10 (3.03) | 1,229 | 1.40 (0.36) | 892 | 3.09 (0.75) | 892 | 1.39 (0.63) | 1,209 | 3.31 (0.55) | 1,226 | 1.67 (2.23) | 1,230 | 3.2 (0.46) | 1,226 |
| W8 | 7.69 (2.86) | 1,210 |  |  |  |  | 1.43 (0.68) | 1,204 | 3.34 (0.53) | 1,211 | 1.47 (2.20) | 1,213 | 3.24 (0.45) | 1,211 |
| W9 | 7.49 (2.79) | 1,201 |  |  |  |  | 1.45 (0.68) | 1,194 | 3.39 (0.53) | 1,201 | 1.51 (2.26) | 1,204 | 3.27 (0.45) | 1,201 |
| W10 | 7.24 (2.62) | 1,175 |  |  |  |  | 1.44 (0.66) | 1,163 | 3.43 (0.52) | 1,173 | 1.51 (2.20) | 1,178 | 3.31 (0.44) | 1,174 |
| W11 | 7.42 (2.68) | 1,130 |  |  |  |  | 1.39 (0.61) | 1,119 | 3.44 (0.52) | 1,128 | 1.54 (2.25) | 1,131 | 3.28 (0.42) | 1,128 |
| *Note*. W# = Wave #, M = Mean, SD = Standard Deviation, RPI = Resistance to Peer Influence, PSMI = Psychosocial Maturity | | | | | | | | | | | | | | |

| Table S2 | | | |
| --- | --- | --- | --- |
| *Baseline Predictors of the Atypical Lying Trajectory* | | | |
|  | Intercept Factor  Std Est (SE) | Slope Factor  Std Est (SE) | Quadratic Factor  Std Est (SE) |
| Parental Hostility | 0.12 (0.13) | -0.09** (0.03) | 0.01 (0.01) |
| Parental Warmth | -0.06 (0.10) | 0.00 (0.02) | 0.00 (0.01) |
| Antisocial Peer Influence | 0.18* (0.09) | -0.03 (0.02) | 0.00 (0.01) |
| Resistance to Peer Influence | -0.28* (0.11) | 0.06* (0.02) | -0.02** (0.01) |
| Perceived Thrill of Crime | 0.10** (0.03) | -0.01 (0.01) | 0.00 (0.00) |
| Psychosocial Maturity | -0.83** (0.14) | 0.04 (0.03) | -0.00 (0.01) |
| Psychopathic Traits | 0.02* (0.01) | -0.00 (0.00) | 0.00 (0.00) |
| Exposure to Violence | -0.00 (0.02) | -0.00 (0.01) | -0.01* (0.00) |
| Neighborhood Condition | 0.01 (0.09) | 0.03 (0.02) | -0.01 (0.01) |
| Sex | -0.74** (0.15) | 0.01 (0.04) | -0.02 (0.01) |
| White | 0.74** (0.17) | -0.02 (0.04) | -0.02 (0.01) |
| Hispanic | 0.08 (0.14) | 0.02 (0.03) | -0.01 (0.01) |
| Other Race | 0.43 (0.29) | 0.02 (0.06) | -0.02 (0.02) |
| *Note*. * p < .05, ** p < .01; N = 1,353; Std Est = Standardized Estimates, SE = Standard Error; Latent growth trajectory was centered at 20 years old; Sex and Race variables are dummy coded with female and black participants as comparison groups, respectively. | | | |

| Table S3 | | | |
| --- | --- | --- | --- |
| *Parallel Process Model of the Atypical Lying Trajectory* | | | |
|  | Atypical Lying Growth Factors | | |
|  | Intercept Factor  Std Est (SE) | Slope Factor  Std Est (SE) | Quadratic Factor  Std Est (SE) |
| Parental Hostility – Intercept Factor | 0.13** (0.03) | 0.00 (0.01) | -0.01* (0.00) |
| Parental Hostility – Slope Factor | 0.00 (0.01) | 0.01** (0.00) | -0.00** (0.00) |
| Parental Warmth – Intercept Factor | -0.34** (0.06) | 0.01 (0.01) | 0.01* (0.01) |
| Parental Warmth – Slope Factor | -0.05** (0.02) | -0.00 (0.00) | 0.00** (0.00) |
| Antisocial Peer Influence – Intercept Factor | 0.37** (0.04) | -0.01* (0.01) | -0.01* (0.00) |
| Antisocial Peer Influence – Slope Factor | -0.01 (0.01) | 0.01** (0.00) | -0.00 (0.00) |
| Antisocial Peer Influence – Quadratic Factor | -0.01* (0.00) | 0.00 (0.00) | 0.00** (0.00) |
| Resistance to Peer Influence – Intercept Factor | -0.34** (0.03) | 0.02* (0.01) | 0.01* (0.00) |
| Resistance to Peer Influence – Slope Factor | -0.00 (0.01) | -0.01** (0.00) | 0.00 (0.00) |
| Resistance to Peer Influence – Quadratic Factor | 0.01** (0.00) | 0.00 (0.00) | -0.00** (0.00) |
| Perceived Thrill – Intercept Factor | 1.51** (0.13) | -0.02 (0.03) | -0.02* (0.01) |
| Perceived Thrill – Slope Factor | -0.02 (0.02) | 0.03** (0.01) | -0.00 (0.00) |
| Perceived Thrill – Quadratic Factor | -0.02* (0.01) | -0.00 (0.00) | 0.00** (0.00) |
| Psychosocial Maturity – Intercept Factor | -0.40** (0.02) | 0.01** (0.01) | 0.01** (0.00) |
| Psychosocial Maturity – Slope Factor | -0.00 (0.01) | -0.01** (0.00) | 0.00 (0.00) |
| Psychosocial Maturity – Quadratic Factor | 0.01** (0.00) | -0.00 (0.00) | -0.00** (0.00) |
| *Note*. * p < .05, ** p < .01; N = 1,353; Std Est = Standardized Estimates, SE = Standard Error; Latent growth trajectory was centered at 20 years old; | | | |

**APPENDIX S2: SUPPLEMENTAL TABLES AND FIGURES**

| Supplemental Table S4 | | | | | | | | | | |
| --- | --- | --- | --- | --- | --- | --- | --- | --- | --- | --- |
| *Pair-wise Pearson Correlation Coefficients of Atypical Lying* | | | | | | | | | | |
|  | Lie 2 | Lie 3 | Lie 4 | Lie 5 | Lie 6 | Lie 7 | Lie 8 | Lie 9 | Lie 10 | Lie 11 |
| Lie 2 | 1 |  |  |  |  |  |  |  |  |  |
| Lie 3 | 0.53** | 1 |  |  |  |  |  |  |  |  |
| Lie 4 | 0.45** | 0.59** | 1 |  |  |  |  |  |  |  |
| Lie 5 | 0.42** | 0.50** | 0.55** | 1 |  |  |  |  |  |  |
| Lie 6 | 0.37** | 0.44** | 0.49** | 0.53** | 1 |  |  |  |  |  |
| Lie 7 | 0.34** | 0.45** | 0.50** | 0.52** | 0.53** | 1 |  |  |  |  |
| Lie 8 | 0.37** | 0.37** | 0.39** | 0.41** | 0.46** | 0.49** | 1 |  |  |  |
| Lie 9 | 0.32** | 0.38** | 0.37** | 0.43** | 0.43** | 0.43** | 0.52** | 1 |  |  |
| Lie 10 | 0.30** | 0.33** | 0.35** | 0.37** | 0.37** | 0.36** | 0.46** | 0.48** | 1 |  |
| Lie 11 | 0.37** | 0.37** | 0.36** | 0.41** | 0.37** | 0.37** | 0.41** | 0.47** | 0.49** | 1 |
| Hos 1 | 0.20** | 0.13** | 0.10** | 0.14** | 0.16** | 0.11** | 0.08** | 0.11** | 0.07* | 0.04 |
| Hos 2 | 0.17** | 0.17** | 0.12** | 0.17** | 0.15** | 0.08* | 0.11** | 0.09** | 0.10** | 0.09** |
| Hos 3 | 0.13** | 0.17** | 0.15** | 0.16** | 0.11** | 0.11** | 0.08* | 0.11** | 0.08* | 0.09** |
| Hos 4 | 0.08* | 0.09** | 0.16** | 0.18** | 0.12** | 0.11** | 0.09** | 0.09** | 0.08* | 0.09** |
| Hos 5 | 0.12** | 0.16** | 0.15** | 0.20** | 0.15** | 0.11** | 0.14** | 0.11** | 0.12** | 0.10** |
| Hos 6 | 0.08* | 0.09** | 0.11** | 0.19** | 0.15** | 0.09** | 0.09** | 0.11** | 0.10** | 0.09** |
| Hos 7 | 0.02 | 0.11** | 0.10** | 0.09* | 0.08* | 0.15** | 0.15** | 0.09** | 0.06 | 0.08* |
| Warm 1 | -0.13** | -0.10** | -0.07* | -0.10** | -0.11** | -0.06* | -0.07* | -0.05 | -0.07* | -0.05 |
| Warm 2 | -0.15** | -0.10** | -0.06 | -0.09** | -0.10** | -0.05 | -0.10** | -0.06 | -0.11** | -0.06 |
| Warm 3 | -0.14** | -0.18** | -0.16** | -0.17** | -0.15** | -0.14** | -0.10** | -0.10** | -0.13** | -0.14** |
| Warm 4 | -0.16** | -0.17** | -0.19** | -0.21** | -0.19** | -0.16** | -0.20** | -0.16** | -0.15** | -0.13** |
| Warm 5 | -0.11** | -0.16** | -0.12** | -0.22** | -0.17** | -0.13** | -0.15** | -0.07* | -0.08* | -0.12** |
| Warm 6 | -0.08* | -0.12** | -0.12** | -0.17** | -0.17** | -0.14** | -0.15** | -0.15** | -0.09** | -0.13** |
| Warm 7 | -0.07 | -0.12** | -0.10** | -0.19** | -0.16** | -0.15** | -0.13** | -0.17** | -0.09** | -0.08* |
| Inf 1 | 0.19** | 0.18** | 0.13** | 0.17** | 0.17** | 0.15** | 0.11** | 0.14** | 0.15** | 0.10** |
| Inf 2 | 0.20** | 0.13** | 0.13** | 0.14** | 0.10** | 0.13** | 0.11** | 0.13** | 0.11** | 0.09** |
| Inf 3 | 0.20** | 0.23** | 0.18** | 0.15** | 0.15** | 0.14** | 0.11** | 0.18** | 0.14** | 0.15** |
| Inf 4 | 0.14** | 0.22** | 0.30** | 0.20** | 0.22** | 0.25** | 0.17** | 0.22** | 0.13** | 0.15** |
| Inf 5 | 0.19** | 0.21** | 0.22** | 0.25** | 0.23** | 0.22** | 0.20** | 0.22** | 0.12** | 0.19** |
| Inf 6 | 0.16** | 0.19** | 0.24** | 0.24** | 0.27** | 0.18** | 0.16** | 0.20** | 0.14** | 0.12** |
| Inf 7 | 0.11** | 0.19** | 0.19** | 0.17** | 0.20** | 0.23** | 0.18** | 0.21** | 0.12** | 0.16** |
| Inf 8 | 0.11** | 0.11** | 0.18** | 0.13** | 0.16** | 0.12** | 0.19** | 0.17** | 0.13** | 0.12** |
| Inf 9 | 0.09** | 0.13** | 0.18** | 0.18** | 0.20** | 0.14** | 0.22** | 0.22** | 0.14** | 0.12** |
| Inf 10 | 0.11** | 0.13** | 0.13** | 0.10** | 0.19** | 0.13** | 0.16** | 0.14** | 0.19** | 0.17** |
| Inf 11 | 0.14** | 0.15** | 0.16** | 0.19** | 0.17** | 0.15** | 0.17** | 0.20** | 0.18** | 0.24** |
| Res 1 | -0.24** | -0.23** | -0.20** | -0.20** | -0.17** | -0.13** | -0.10** | -0.10** | -0.15** | -0.18** |
| Res 2 | -0.23** | -0.20** | -0.20** | -0.20** | -0.13** | -0.13** | -0.05 | -0.12** | -0.12** | -0.14** |
| Res 3 | -0.19** | -0.28** | -0.23** | -0.21** | -0.18** | -0.17** | -0.13** | -0.14** | -0.14** | -0.18** |
| Res 4 | -0.16** | -0.23** | -0.26** | -0.26** | -0.20** | -0.19** | -0.11** | -0.13** | -0.13** | -0.19** |
| Res 5 | -0.14** | -0.19** | -0.19** | -0.27** | -0.23** | -0.22** | -0.12** | -0.18** | -0.15** | -0.18** |
| Res 6 | -0.18** | -0.15** | -0.20** | -0.22** | -0.29** | -0.18** | -0.16** | -0.13** | -0.14** | -0.17** |
| Res 7 | -0.16** | -0.21** | -0.19** | -0.25** | -0.26** | -0.27** | -0.20** | -0.18** | -0.20** | -0.19** |
| Res 8 | -0.14** | -0.17** | -0.16** | -0.21** | -0.21** | -0.23** | -0.22** | -0.23** | -0.20** | -0.21** |
| Res 9 | -0.14** | -0.19** | -0.22** | -0.25** | -0.25** | -0.24** | -0.20** | -0.24** | -0.21** | -0.24** |
| Res 10 | -0.13** | -0.17** | -0.20** | -0.20** | -0.21** | -0.20** | -0.18** | -0.17** | -0.23** | -0.22** |
| Res 11 | -0.18** | -0.19** | -0.20** | -0.22** | -0.19** | -0.18** | -0.17** | -0.20** | -0.23** | -0.25** |
| Thrill 1 | 0.19** | 0.20** | 0.21** | 0.21** | 0.20** | 0.19** | 0.12** | 0.20** | 0.12** | 0.16** |
| Thrill 2 | 0.25** | 0.20** | 0.24** | 0.21** | 0.21** | 0.19** | 0.18** | 0.21** | 0.19** | 0.18** |
| Thrill 3 | 0.23** | 0.25** | 0.26** | 0.24** | 0.21** | 0.21** | 0.17** | 0.22** | 0.21** | 0.21** |
| Thrill 4 | 0.15** | 0.19** | 0.26** | 0.23** | 0.23** | 0.22** | 0.20** | 0.24** | 0.17** | 0.20** |
| Thrill 5 | 0.16** | 0.20** | 0.25** | 0.26** | 0.27** | 0.26** | 0.21** | 0.25** | 0.21** | 0.22** |
| Thrill 6 | 0.19** | 0.22** | 0.26** | 0.25** | 0.31** | 0.24** | 0.25** | 0.29** | 0.19** | 0.17** |
| Thrill 7 | 0.16** | 0.24** | 0.26** | 0.24** | 0.26** | 0.30** | 0.26** | 0.27** | 0.22** | 0.21** |
| Thrill 8 | 0.14** | 0.20** | 0.24** | 0.21** | 0.20** | 0.21** | 0.22** | 0.29** | 0.23** | 0.22** |
| Thrill 9 | 0.13** | 0.18** | 0.20** | 0.20** | 0.20** | 0.15** | 0.20** | 0.27** | 0.18** | 0.20** |
| Thrill 10 | 0.14** | 0.16** | 0.19** | 0.18** | 0.20** | 0.17** | 0.20** | 0.26** | 0.25** | 0.24** |
| Thrill 11 | 0.20** | 0.19** | 0.20** | 0.20** | 0.20** | 0.17** | 0.22** | 0.23** | 0.24** | 0.24** |
| Mat 1 | -0.22** | -0.23** | -0.20** | -0.23** | -0.21** | -0.18** | -0.18** | -0.19** | -0.18** | -0.18** |
| Mat 2 | -0.34** | -0.30** | -0.28** | -0.25** | -0.23** | -0.20** | -0.20** | -0.20** | -0.19** | -0.22** |
| Mat 3 | -0.27** | -0.37** | -0.29** | -0.26** | -0.27** | -0.23** | -0.21** | -0.22** | -0.21** | -0.20** |
| Mat 4 | -0.24** | -0.30** | -0.42** | -0.30** | -0.30** | -0.26** | -0.21** | -0.25** | -0.19** | -0.21** |
| Mat 5 | -0.21** | -0.30** | -0.29** | -0.37** | -0.35** | -0.34** | -0.24** | -0.26** | -0.18** | -0.23** |
| Mat 6 | -0.15** | -0.25** | -0.26** | -0.31** | -0.45** | -0.32** | -0.26** | -0.25** | -0.19** | -0.22** |
| Mat 7 | -0.15** | -0.29** | -0.27** | -0.30** | -0.34** | -0.43** | -0.27** | -0.28** | -0.22** | -0.24** |
| Mat 8 | -0.17** | -0.19** | -0.18** | -0.25** | -0.28** | -0.29** | -0.37** | -0.27** | -0.22** | -0.24** |
| Mat 9 | -0.18** | -0.22** | -0.26** | -0.28** | -0.29** | -0.29** | -0.30** | -0.38** | -0.28** | -0.29** |
| Mat 10 | -0.19** | -0.23** | -0.23** | -0.24** | -0.27** | -0.26** | -0.26** | -0.30** | -0.36** | -0.32** |
| Mat 11 | -0.24** | -0.22** | -0.22** | -0.26** | -0.27** | -0.25** | -0.27** | -0.29** | -0.33** | -0.44** |
| Note: Formatting of the left column uses the following pattern: variable followed by wave number; Lie = Atypical Lying, Hos = Parental Hostility, Warm = Parental Warmth; Inf = Antisocial Peer Influence; Res = Resistance to Peer Influence, Thrill = Perceived Thrill of Crime; Mat = Psychosocial Maturity | | | | | | | | | | |

| Supplemental Table S5 | | | | | | | | | | | | | | | | |
| --- | --- | --- | --- | --- | --- | --- | --- | --- | --- | --- | --- | --- | --- | --- | --- | --- |
| *Longitudinal Measurement Invariance Across Waves* | | | | | | | | | | | | | | | | |
| Construct | Invariance Level | CFI | TLI | | | RMSEA | SRMR | | Δ CFI | | Δ TLI | | Δ RMSEA | | Δ SRMR | |
| Atypical Lying (Waves 2-11) | | | | | | | | | | | | | | | | |
|  | Configural | 0.997 | 0.998 | | | 0.048 | 0.024 | | - | | - | | - | | - | |
|  | Metric | 0.998 | 0.998 | | | 0.042 | 0.029 | | +0.001 | | 0 | | -0.006 | | +0.005 | |
|  | Scalar | 0.999 | 0.998 | | | 0.026 | 0.025 | | +0.001 | | 0 | | -0.016 | | -0.004 | |
| Parental Hostility (Waves 1-7) | | | | | | | | | | | | | | | | |
|  | Configural | 0.979 | 0.983 | | | 0.059 | 0.099 | | - | | - | | - | | - | |
|  | Metric | 0.980 | 0.981 | | | 0.059 | 0.105 | | +0.001 | | -0.002 | | 0 | | +0.006 | |
|  | Scalar | 0.982 | 0.980 | | | 0.056 | 0.102 | | +0.002 | | -0.001 | | -0.003 | | -0.003 | |
| Parental Warmth (Waves 1-7) | | | | | | | | | | | | | | | | |
|  | Configural | 0.998 | 0.999 | | | 0.066 | 0.028 | | - | | - | | - | | - | |
|  | Metric | 0.998 | 0.998 | | | 0.066 | 0.031 | | 0 | | -0.001 | | 0 | | +0.003 | |
|  | Scalar | 0.999 | 0.999 | | | 0.049 | 0.028 | | +0.001 | | +0.001 | | -0.017 | | -0.003 | |
| Antisocial Peer Influence (Waves 1-11) | | | | | | | | | | | | | | | | |
|  | Configural | 0.993 | 0.995 | | | 0.100 | 0.054 | | - | | - | | - | | - | |
|  | Metric | 0.994 | 0.994 | | | 0.092 | 0.060 | | +0.001 | | -0.001 | | -0.008 | | +0.006 | |
|  | Scalar | 0.997 | 0.995 | | | 0.064 | 0.054 | | +0.003 | | +0.001 | | -0.028 | | -0.006 | |
| Resistance to Peer Influence (Waves 1-11) | | | | | | | | | | | | | | | | |
|  | Configural | 0.984 | 0.987 | | | 0.051 | 0.046 | | - | | - | | - | | - | |
|  | Metric | 0.984 | 0.984 | | | 0.051 | 0.052 | | 0 | | -0.003 | | 0 | | +0.006 | |
|  | Scalar | 0.989 | 0.985 | | | 0.043 | 0.048 | | +0.005 | | +0.001 | | -0.008 | | -0.004 | |
| Perceived Thrill of Crime (Waves 1-11) | | | | | | | | | | | | | | | | |
|  | Configural | 0.891 | | 0.928 | 0.160 | | | 0.037 | | - | | - | | - | | - |
|  | Metric | 0.919 | | 0.925 | 0.138 | | | 0.048 | | +0.028 | | -0.003 | | -0.022 | | +0.011 |
|  | Scalar | 0.915 | | 0.925 | 0.141 | | | 0.048 | | -0.004 | | 0 | | +0.003 | | 0 |
| Psychosocial Maturity (Waves 1-11) | | | | | | | | | | | | | | | | |
|  | Configural | 0.987 | | 0.988 | 0.063 | | | 0.053 | | - | | - | | - | | - |
|  | Metric | 0.986 | | 0.986 | 0.066 | | | 0.057 | | -0.001 | | -0.002 | | +0.003 | | +0.004 |
|  | Scalar | 0.988 | | 0.987 | 0.060 | | | 0.053 | | +0.002 | | +0.001 | | -0.006 | | -0.004 |
| *Note*. Δ = Change in fit statistic between Configural and Metric invariance or between Metric and Scalar invariance | | | | | | | | | | | | | | | | |

| Supplemental Table S6 | | | |
| --- | --- | --- | --- |
| *Unconditional Latent Growth Trajectory Fit Statistics* | | | |
|  | AIC | BIC | SABIC |
| Atypical Lying (Waves 2-11, N = 1,334) | | | |
| Intercept only | 56849.09 | 56906.25 | 56871.30 |
| Linear | 56078.92 | 56151.67 | 56107.19 |
| **Quadratic** | **56002.56** | **56096.09** | **56038.91** |
| Parental Hostility (Waves 1-7, N = 1,347) | | | |
| Intercept only | 6554.32 | 6569.94 | 6560.41 |
| **Linear** | **6215.10** | **6246.34** | **6227.28** |
| Quadratic | 6200.00 | 6252.05 | 6220.29 |
| Parental Warmth (Waves 1-7, N = 1,347) | | | |
| Intercept only | 13595.74 | 13611.35 | 13601.83 |
| **Linear** | **13406.46** | **13437.69** | **13418.64** |
| Quadratic | 13380.51 | 13432.57 | 13400.80 |
| Antisocial Peer Influence (Waves 1-11, N = 1,353) | | | |
| Intercept only | 26325.84 | 26341.47 | 26331.94 |
| Linear | 25938.66 | 25969.92 | 25950.86 |
| **Quadratic** | **25850.53** | **25902.64** | **25870.87** |
| Resistance to Peer Influence (Waves 1-11, N = 1,353) | | | |
| Intercept only | 18730.76 | 18746.39 | 18736.86 |
| Linear | 17011.29 | 17042.55 | 17023.49 |
| **Quadratic** | **16792.80** | **16844.90** | **16813.14** |
| Perceived Thrill (Waves 1-11, N = 1,353) | | | |
| Intercept only | 55805.94 | 55821.57 | 55812.04 |
| Linear | 55025.78 | 55057.04 | 55037.98 |
| **Quadratic** | **54841.22** | **54893.32** | **54861.56** |
| Psychosocial Maturity (Waves 1-11, N = 1,353) | | | |
| Intercept only | 13291.91 | 13307.54 | 13298.01 |
| Linear | 12298.29 | 12329.55 | 12310.49 |
| **Quadratic** | **12129.08** | **12181.18** | **12149.41** |
| *Note*. Bolded is best fit model, AIC = Akaike Information Criterion, BIC = Bayesian Information Criterion, SABIC = Sample-Size Adjusted BIC | | | |

| Supplemental Table S7 |  |  |  |
| --- | --- | --- | --- |
| *Unconditional Latent Trajectory Growth Factor Covariances* | | | |
|  | Intercept Factor  Std Est (SE) | Slope Factor Std Est (SE) | Quadratic Factor Std Est (SE) |
| Atypical Lying (Waves 2-11, N = 1,334) | | | |
| Intercept Factor | - | -0.12** (0.03) | -0.05** (0.00) |
| Slope Factor |  |  | -0.01* (0.00) |
| Parental Hostility (Waves 1-7, N = 1,347) | | | |
| Intercept Factor | - | 0.01** (0.00) |  |
| Parental Warmth (Waves 1-7, N = 1,347) | | | |
| Intercept Factor | - | 0.06** (0.01) |  |
| Antisocial Peer Influence (Waves 1-11, N = 1,353) | | | |
| Intercept Factor | - | -0.00 (0.00) | -0.00** (0.00) |
| Slope Factor |  |  | 0.00** (0.00) |
| Resistance to Peer Influence (Waves 1-11, N = 1,353) | | | |
| Intercept Factor | - | 0.00 (0.00) | -0.00** (0.00) |
| Slope Factor |  |  | 0.00* (0.00) |
| Perceived Thrill (Waves 1-11, N = 1,353) | | | |
| Intercept Factor | - | -0.01 (0.02) | -0.01** (0.01) |
| Slope Factor |  |  | -0.00 (0.00) |
| Psychosocial Maturity (Waves 1-11, N = 1,353) | | | |
| Intercept Factor | - | -0.00** (0.00) | -0.00** (0.00) |
| Slope Factor |  |  | 0.00 (0.00) |
| *Note*. * p < .05, ** p < .01; Std Est = Standardized Estimates, SE = Standard Error | | | |

**Supplemental Figure 1**

*Unconditional Univariate Quadratic Growth of Atypical Lying Features*


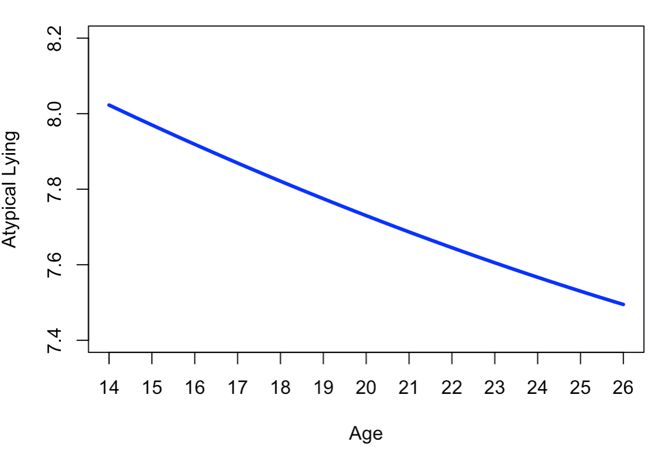


**Supplemental Figure 2**

*Unconditional Univariate Linear Growth of Parental Hostility*

**
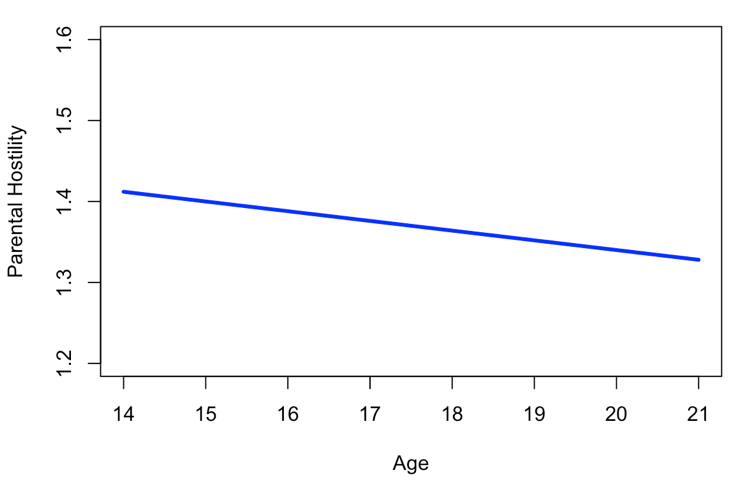
**

**Supplemental Figure 3**

*Unconditional Univariate Linear Growth of Parental Warmth*

**
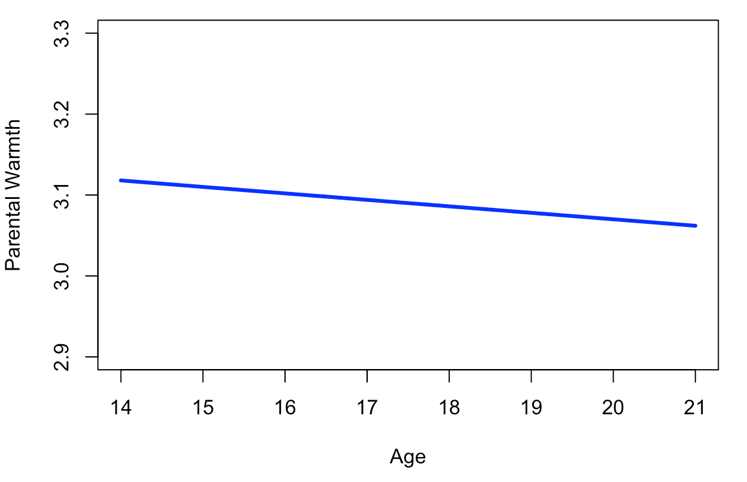
**

**Supplemental Figure 4**


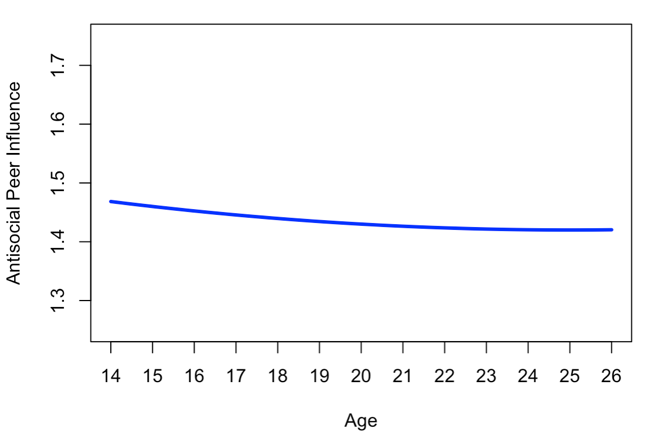
*Unconditional Univariate Quadratic Growth of Antisocial Peer Influence*

**Supplemental Figure 5**


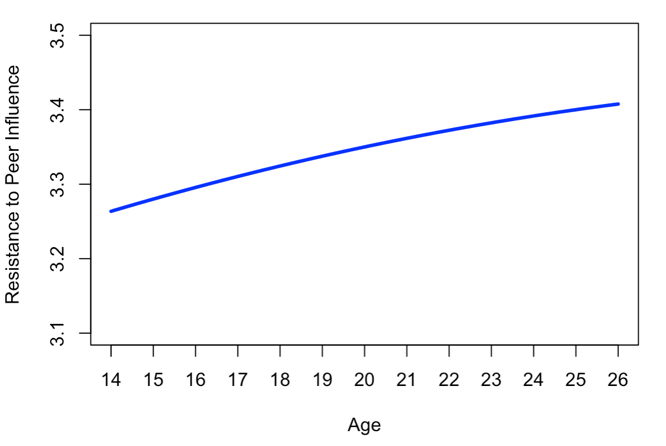
*Unconditional Univariate Quadratic Growth of Resistance to Peer Influence*

**Supplemental Figure 6**


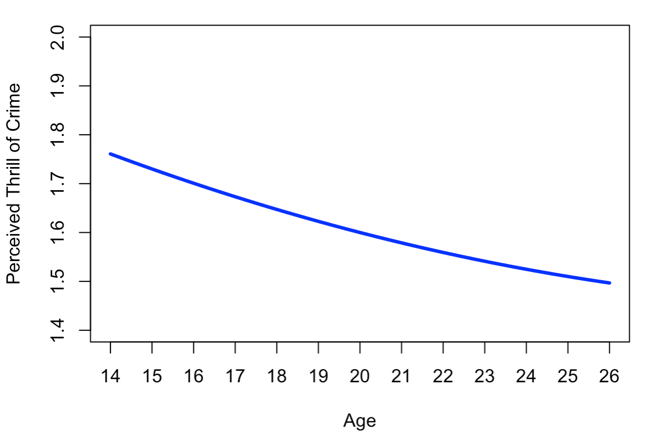
*Unconditional Univariate Quadratic Growth of Thrill of Crime*

**Supplemental Figure 7**


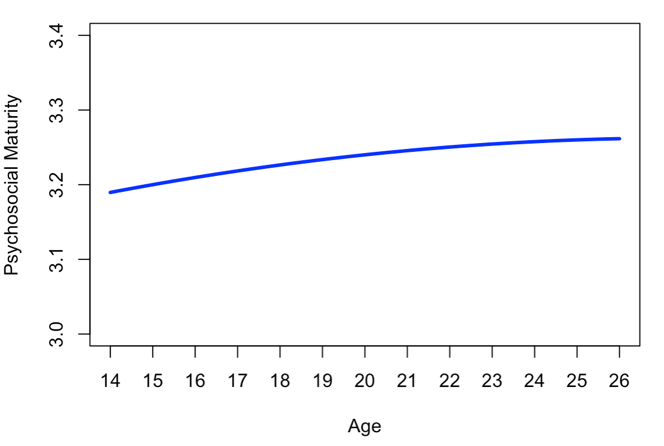
*Unconditional Univariate Quadratic Growth of Psychosocial Maturity*
